# Emerging Trends and Recent Progress of MXene as a Promising 2D Material for Point of Care (POC) Diagnostics

**DOI:** 10.3390/diagnostics13040697

**Published:** 2023-02-12

**Authors:** Raghuraj Singh Chouhan, Maitri Shah, Drishya Prakashan, Ramya P R, Pratik Kolhe, Sonu Gandhi

**Affiliations:** 1 Department of Environmental Sciences, Institute “Jožef Stefan”, Jamova 39, 1000 Ljubljana, Slovenia; 2 DBT-National Institute of Animal Biotechnology (NIAB), Hyderabad 500032, India; 3 RCB-Regional Centre for Biotechnology, Faridabad 121001, India

**Keywords:** MXenes, 2D nanomaterials, biosensors, POC testing

## Abstract

Two-dimensional (2D) nanomaterials with chemical and structural diversity have piqued the interest of the scientific community due to their superior photonic, mechanical, electrical, magnetic, and catalytic capabilities that distinguish them from their bulk counterparts. Among these 2D materials, two-dimensional (2D) transition metal carbides, carbonitrides, and nitrides with a general chemical formula of M_n+1_X_n_T_x_ (where *n* = 1–3), together known as MXenes, have gained tremendous popularity and demonstrated competitive performance in biosensing applications. In this review, we focus on the cutting-edge advances in MXene-related biomaterials, with a systematic summary on their design, synthesis, surface engineering approaches, unique properties, and biological properties. We particularly emphasize the property–activity–effect relationship of MXenes at the nano–bio interface. We also discuss the recent trends in the application of MXenes in accelerating the performance of conventional point of care (POC) devices towards more practical approaches as the next generation of POC tools. Finally, we explore in depth the existing problems, challenges, and potential for future improvement of MXene-based materials for POC testing, with the goal of facilitating their early realization of biological applications.

## 1. Introduction

Major advancements in the health-care industry and subsequent analytical industry have been centered on the fabrication of compact, reusable, and efficient miniature platforms or point of care (POC) solutions. POC testing (POCT) is a discipline that strives to develop diagnostic techniques that provide a number of benefits, including the potential to deliver quick and reliable results, easy operation, cost-effectiveness, and a lack of specialized equipment. Biosensors are devices that are used to detect target molecules with high sensitivity and specificity. Needless to say, the effectiveness and performance of these biosensor POC devices are heavily reliant on the quality of the material that makes up the device [1,2,3,4,5]. Biosensing materials have a lengthy history, comprising a diverse variety of 0D, 1D, and 2D nanomaterials such as transition metal nanoparticles [6], gold nanoparticles [7], nanorods [8,9,10], and MoSe_2_ [11,12]. Because of its excellent potential, MXene is among the greatest materials for the fabrication of biosensors among recent nanomaterials drawing attention. Several breakthroughs in the last few years have greatly enhanced the synthesis of new MAX phases with ordered double-transition metals and as a result the synthesis of novel MXenes with diversified chemical and structural complexity, which is rarely observed in other families of two-dimensional (2D) materials [13]. MXenes and their derivatives are currently well known in the realm of biosensing and have exceptional sensitivity, stability, range of detection, and low detection limit [14,15]. MXenes are two-dimensional inorganic compounds with a number of atomic layers that are comprised of transition metal in combination with carbon, nitrogen, or both, such as titanium carbide (Ti_3_C_2_) and titanium carbonitride (Ti_2_CN), endowing them with exceptional features, such as high conductivity and superior fluorescent, optical, and plasmonic properties [16,17]. They have the general formula M_n+1_X_n_T_x_, where M represents early transition metals, X represents carbon or nitrogen, T represents surface functional groups (-OH, F, =O), and n represents an integer (n = 1–3). They are made by selectively etching closely packed multilayered MAX phases with alternate layers of M and A that have strong M-X bonds and weak M-A bonds. Because of their distinct surface chemistry and intriguing electrochemical behavior, as well as their excellent biocompatibility, they are perfect as a solid support for the fabrication of cutting-edge electrochemical sensing and biosensing devices [18]. MXene has recently been employed successfully in a variety of sensing purposes, targeted drug delivery, cancer therapeutics, energy storage [19], heat-resistant material production, catalysis [20], and many more. In spite of their versatility and superior performance in biosensing applications, MXenes face a number of obstacles, including the unavoidable use of hazardous chemicals and laborious etching methods. Furthermore, present synthetic processes have difficulty in scaling up yields and managing characteristics such as size, surface termination, and flaws in the resultant MXenes [21]. Given the fast growth of MXene-based research and technology, it is important to update our current understanding on different properties and future applications. Hence, in this review we focus on the most recent breakthroughs in MXene-related biomaterials, providing a systematic overview of their design, synthesis, surface engineering methodologies, unique features, and biological consequences. We review recent trends in the use of MXenes to accelerate the performance of conventional POC devices towards more practical approaches as the next generation of POC equipment. Finally, we put forth a critical analysis of the current problems, limitations, and prospects for the future enhancement of MXene-based materials for POC testing, with an emphasis on their enormous sensing potential, which is yet to be unearthed.

## 2. Design, Synthesis, and Surface Functionalization of MXenes

**Figure 1:**
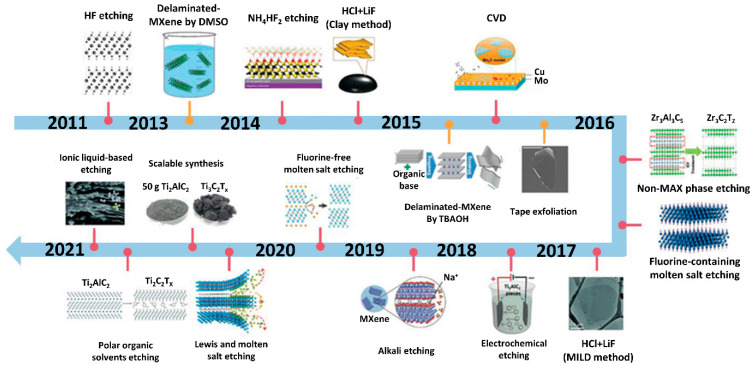
Evolution of the synthesis of MXene employing different approaches. (Reprinted with copyright permission from [23] by John Wiley and Sons and Copyright Clearance Center).

Electrical properties, physicochemical traits, and a variety of applications of MXenes were significantly influenced by the synthesis methods of MXenes. More than 20 MXenes have been produced by selective chemical etching of a few atomic layers acquired from pretreatment agents such as carbide, nitride, and carbonitride (Figure 1). There are mainly three approaches for MXene synthesis: top-down, bottom-up, and etching [22]. A brief introduction of all approaches is given below.

### 2.1. Top-Down Approach

In this method, large or huge material is exfoliated into smaller sheets, which could be monolayered or single-layer. Bulk precursors might be crystalline in nature, which will be converted into sheets. Methods such as ball milling [24], liquid exfoliation, hydrothermal/solvent-assisted heating, ultrasonication, and microwave-assisted exfoliation are used in a top-down approach. The top-down approach is appropriate for conditions such as acid reflux or chemical etching. Moreover, this method is simple to implement and is used to generate a large amount of material. However, there are difficulties, such as the limited yield and the necessity for certain treatments.

#### 2.1.1. Hydrothermal Method

The hydrothermal method is an inhomogeneous reaction process that requires heating aqueous solutions over the water’s boiling point in a high-pressure autoclave that also involves precursor material. The hydrothermal method is an effective method for creating multipatterned, two-dimensional MXene components while being environmentally friendly. Afterward, by tuning the conditions of the hydrothermal method, physical properties such as size, shape, and thickness can be manipulated. Xue et al. synthesized Ti_3_C_2_ MXene quantum dots (MQDs) with the help of the facile hydrothermal method, and by managing the temperature conditions, the size of the luminescent QD was also controlled. With an elevation in temperature, the size and thickness of QDs decreases [25]. The hydrothermal method is an efficient way to create multipatterned, 2D MXene components without using hazardous acids, such as HF. Using a novel leaching technique without fluorine, by dipping Ti_3_AlC_2_ in an aqueous alkali solution at roughly 85 °C for 100 h and then hydrothermally treating it with 1 M H_2_SO_4_ at 85 °C for 1.5 h, aluminum layers made with the MAX system are produced. When—instead of an aqueous solution—any organic solution is preferred for a reaction, it is called a “solvothermal reaction.” MXene precursors are more miscible than aqueous phases, and in organic phases of crystalline nature, particle distribution is more controllable than in the hydrothermal method [26].

#### 2.1.2. Ball-Milling Method

For the synthesis of 0D QDs, the ball-milling approach has been extensively employed for the top-down method. A variety of variables, such as speed and timing of milling, the amount of powder used for milling, and dry/wet type of milling, are responsible for the physical characteristics of nanomaterials. Zhang et al., using Ti_3_C_2_T_x_ where T can be O, OH, or F, demonstrated that MXene size can be reduced from micrometers to approximately 6 nm nanodots by a ball-milling method with red phosphorus [27].

#### 2.1.3. Ultrasonication Method

**Figure 2:**
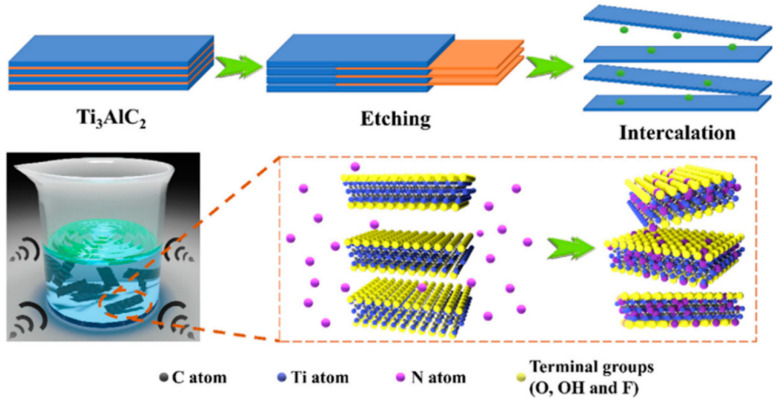
Stepwise procedure for ultrasound-assisted synthesis of N-doped MXene. (Reprinted from [29] with copyright permission for figure obtained from Elsevier).

Ultrasonication is an eco-friendly, nonhazardous method. Solvent acoustics, cavitation, and reverberation cause changes in a layered material. Organic solvents such as dimethyl sulfoxide (DMSO), dimethyl formamide (DMF), N-methyl-2-pyrrolidone (NMP), and tetrabutylammonium hydroxide (TBAOH) are used for ultrasound-assisted synthesis [28]. Nitrogen-doped MXene (Figure 2) was synthesized by ultrasonication, in which MXene was prepared by fluoride etching followed by ultrasonication of the mixture of MXene and ammonia [29].

### 2.2. Bottom-Up Approach

In this approach, molecular material is used as a starting material, unlike the bulk used in the top-down method. The involvement of molecular or tiny precursors, an increase in atomic usage, tunable structural and functional features, and the ability to perform functionalization more quickly are all advantages of a bottom-up approach over a top-down approach [30]. Bottom-up synthesis is simpler than top-down synthesis because it only requires one pot reaction; however, more research on the bottom-up synthesis protocol is required [31].

#### 2.2.1. Molten Salt Synthesis

**Figure 3:**
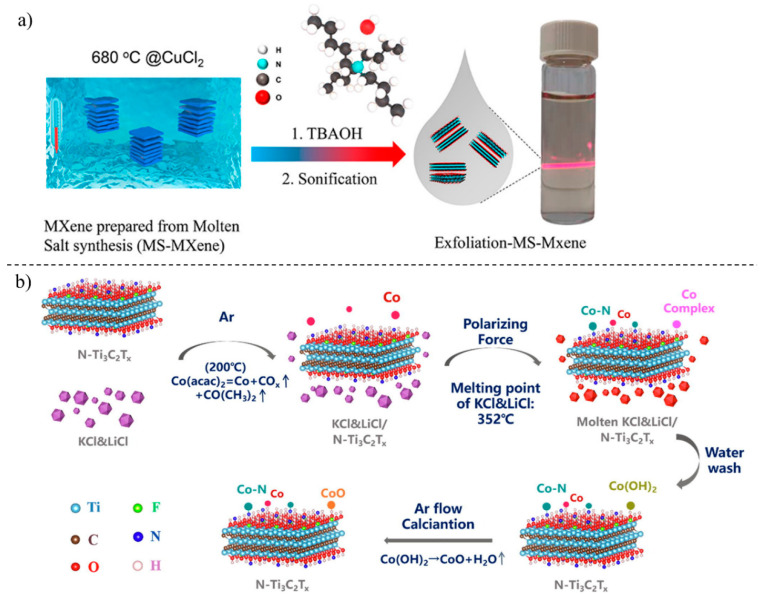
MXene synthesis by molten salt method. (**a**) Molten calcium chloride exfoliated Mxene synthesis; adapted with permission [33]. Copyright (2021) American Chemical Society. (**b**) N-doped cobalt modified MXene sheets with molten salt mixture. (Reprinted from [34] with copyright permission for figure obtained from Elsevier).

In molten salt synthesis, molten salt is used as a reaction medium containing a precursor of nanomaterials. Molten salt enhances reaction kinetics by depleting the distance between the reacting species and acting as a solvent in the reaction. In the synthesis of molybdenum carbide nanodots coupled with carbon nanosheets (Mo_2_C/C), molten NaCl solution, sucrose and Mo precursor was used. The mixture was calcinated at around 800 °C for 2 h, and Mo and sucrose were confined between the nanocrystals of NaCl [32]. Cl^−^-functionalized MXene sheets were obtained with molten salt-assisted sonication followed by functionalization after exfoliation by TBAOH (tetrabutylammonium hydroxide) [33]. Further, molten salt at high temperatures has been used to obtain multivalent vacancy in MXenes (Figure 3) [34].

#### 2.2.2. Pyrolysis Method

In a bottom-up approach, pyrolysis is a practicable method for MXene synthesis that is both simple and environmentally friendly. As the increased interest in the bottom-up approach resulted in the application of different molecular precursors and optimized conditions for the synthesis of MXenes, advancement in the bottom-up approach could be observed in the pyrolysis method, i.e., efficiently performed, simple procedure, high concentration of monodispersed product, high yield, and amended crystallinity in the product. Wang and his group synthesized MXene nanocomposites by employing the pyrolysis technique [35].

### 2.3. Etching Method

Etching is the removal of the surface layer in fabrication with the help of chemicals. There are several methods for preparing MXenes. Different terminal functions could be added to the metal atoms or central atom to complete their coordination spheres and reduce their surface Gibbs free energy as a result of modifications in their etching techniques. The MXene’s surface characteristics therefore have a significant impact on their manufacture.

#### 2.3.1. Hydrofluoric Acid (HF) Etching

**Figure 4:**
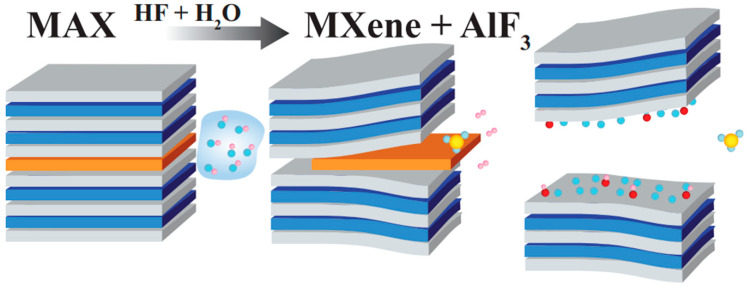
Exfoliation of Ti_3_AlC_2_ MAX phase using HF/H_2_O. (Reprinted with permission from [38] Copyright (2016) American Chemical Society).

Hydrofluoric acid (HF) is known as the fluoride solution in water. HF etching is a commonly practiced protocol for MXene synthesis. HF causes strong irritation in the MAX phase. In this method, reaction time, temperature applied, and strength of fluoride (F-) ions are important variables for the quality of MXene. Using the HF etching method, distinct functionalities such as (-OH, -O, -F) can be imparted to the MXenes surface. HF implies regular displacement mechanism, when Ti_3_AlC_2_ phase treated with the HF solution with evolution of H_2_ gas confiscate Al layers from the phase [36]. A series of the MAX complexes of Ti_2_AlC, (Ti_0.5_Nb_0.5_) 2AlC, Ti_3_AlCN, Ta_4_AlC_3_, (V_0.5_Cr_0.5_) 3AlC_2_, Nb_2_AlC, Zr_3_Al_3_C_5_, Ti_3_SiC_2_, and Mo_2_Ga_2_C have been effectively converted into MXenes using the HF acid etching method [37]. Srivastava et al. showed synthesis of Ti_3_C_2_ through exfoliation of Ti_3_AlC_2_ with HF treatment (Figure 4) [38].

#### 2.3.2. Modified Acid Etching

Even though it is commonly used and has good outcomes, because of the poisonous properties and hazardous effects of HF, direct treatment with HF is replaced by fluoride salts such as LiF, NH_4_HF_2_, FeF_3_, KF, and NaF. HF treatment with MAX phases containing Al or Ga gives the unwanted by-product of hydrated fluorides (i.e., AlF_3_·3H_2_O) [39]. By changing the method, one can avoid this. Another advantage of the modified method over regular HF etching is cation chelation, which decreases interlayer force [40].

#### 2.3.3. Modified Fluoride-Based Etching

**Figure 5:**
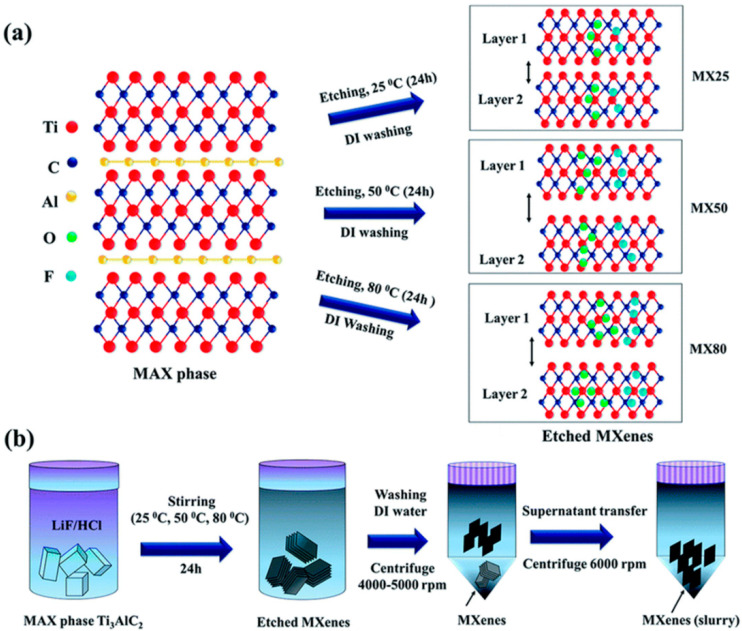
(**a**) Effect of temperature on etching; (**b**) Ti_3_AlC_2_ synthesis protocol (Reprinted from [41] with permission from the Royal Society of Chemistry).

Researchers have studied hard to identify improved outcomes for the removal of atoms from MAX layers to avoid the toxicity caused by HF etching. Aside from HF, fluoride salts such as KF, NaF, LiF, and NH_4_F are used as fluoride precursors with the addition of other strong acids, such as HCl. In this method, the ratio of fluoride to acid strength plays a role in the synthesis of MXene sheets with controllable size. Kumar et al. studied the effect of temperature on etching with fluoride salt and acid LiF/HCl, in which they observed that etching efficiency increases with increasing temperature (Figure 5) [41].

#### 2.3.4. Molten Salt Etching

Similar to the bottom-up approach, molten salt solutions are used for the etching and delamination of MXenes. In this method, a mixture of fluoride salts (LiF, NaF, and KF) has been used at high temperatures. The molten salt method has a faster reaction time and can be synthesized to a limited degree. Along with the difficulties of this method, etching requires a considerable amount of heat and energy. The final product has lower purity and a less crystalline nature, resulting in the production of final MXene with numerous significant defects and vacancies [42].

#### 2.3.5. Etching without Fluorine-Based Species

Most of the synthetic approaches for MXene are based on HF or fluoride-based salt and acid mixtures. This procedure generates -O, -F functionalization at the surface and interfaces. Fluoride-based MXene showed depletion of electrochemical properties [43]. As a result, fluoride-free synthesis techniques are required to improve the electrochemical properties. Etching and delamination of Al layers with strong alkali NaOH were used as an etching agent [44].

Surface functionalization, or interface functionalization, is responsible for the physical and chemical properties that could be used for various applications. Different functional groups—oxygen, fluorine, and hydroxyl—can be rendered onto MXene layers. MXene bears combinations of different functional groups that result from various synthetic approaches [45]. Techniques such as neutron scattering images and nuclear magnetic resonance spectroscopy have been utilized to validate surface modification and atomic distribution. Through a hydrophilic surface, ionic/polar species can be adsorbate. It has been noted that F-group compounds are often used when MXenes are used for adsorptive purposes. Since hydroxyl and oxygen groups are intended to be considerably more stable, fluorine group terminations can be made up for after washing or keeping in water using OH groups. Because of this, -O and -OH functional groups are involved in a variety of terminations for MXenes that are made possible by modifying chemical etching techniques [46]. Recently, the idea of chemical vapor deposition technology has been utilized purposefully to manufacture bare MXene (MO_2_C) without the attachment of functional groups [47]. They are outstanding in terms of their physical characteristics, which have also been effectively investigated using density functional theory (DFT). They are particularly active and have more chemical reactivity than other constituents, as shown by the molecules that do not undergo termination. Combining two or more of the methods discussed above can also be used to synthesize and functionalize MXenes. A heterojunction may also be used to describe the combination used. Future research should concentrate on the adsorptive properties of MXenes without regard to functional group connections [48]. Table 1 represents the methods used for the synthesis and functionalization of MXene.

## 3. Properties and Biological Effects of MXenes

Excellent Young’s modulus, heat and electron transfer, as well as a tunable band gap are some of the phenomenal MXene characteristics. Remarkably, MXenes are unique among 2D nanomaterials, including graphene, owing to their hydrophilic exterior and strong metal-like conductivity (Table 2) [60,61,62]. Last but not least, their composition (such as the generation of homogeneous mixtures of various transition metal and carbon or nitrogen elements), surface modification (via chemical and heat manipulations), and architecture/arrangement modifications can all be utilized to adjust their characteristics and applicability [63,64]. The principal characteristics of the MXene series are listed below.

### 3.1. Electrical Properties

The electron transfer characteristics of MXenes are one of the most important aspects of significance, and can be tailored by modifying the functionalization moieties, adjusting the stoichiometry, or producing a solid-state solution. The electron transport properties of MXene pressed disks were comparable to graphene (coefficient of friction ranging from 22 Ω to 339 Ω, depending on the chemical composition) and greater than CNTs and rGO [95,96]. Surface alteration by providing heat and basic treatment is a valuable technique for enhancing electronic characteristics. Kim et al. reported twofold increased thermoelastic properties of 2D molybdenum-based MXenes due to modification in functional groups (either addition or removal) and eventual change in embedded surface groups [97].

### 3.2. Mechanical Properties

Carbon and nitrogen form very stable and strong bonds with metal, leading to exceptional mechanical properties of MXenes. Some simulation-based studies revealed a higher elastic property of MXene than their native MAX phase. As a result of the presence of various functional groups, MXenes intercalated with polymeric matrices are more effective than graphene for use as composite materials [98,99]. Titanium based MXenes exhibited a hydrophilic nature with a low contact angle compared to graphene [100]. Additionally, it was observed that the Young modulus of MXene (both C and N) lowers with every added layers [101]. Even though measurement techniques can be hard, the lack of control over MXene surface modifications, the occurrence of intrinsic defects (such as gaps), and limited composite integrations are still a bigger problem and make it hard to evaluate MXenes’ mechanical properties [102].

### 3.3. Thermal Properties

Due to their ongoing downsizing, MXenes are essential for electrical and energy-related thermal dispersion technologies [103]. Simulations projected reduced heat contraction constants and greater heat conductivities compared to other monolayered compounds [104]. Some titanium-, zirconium-, and strontium-based MXenes display thermal conductivities in the range of 22 to 472 Wm^−1^ K^−1^ at room temperature [105]. Heat conductivity of compounds eliminated the oxygen rise with respect to atomic number of the associated metal [106]. Finally, the relationship between size of particles and heat transfer capacity emphasizes the significance of morphological regulation and modification in MXene production.

### 3.4. Magnetic Properties

Unlike MAX phases, investigations on MXenes’ magnetic characteristics have been extended owing to the magnetic possibilities. The presence of magnetic properties has been hypothesized for a number of pure compounds, including carbides and nitrides of titanium, iron, zirconium, chromium and zirconium: Ti_4_C_3_ [107], Ti_3_CN [108], Fe_2_C [109], Cr_2_C [110], Ti_3_N_2_ [111], Ti_2_N [112], Zr_2_C, and Zr_3_C_2_ [113]. However, when dealing with terminations, separate analyses involving individual MXenes and chemical modification class are required. Examples include the fact that functional groups inhibit the magnetic properties for Ti_3_CNT_x_ and Ti_4_C_3_T_x_, but conserve the ferromagnetic properties for Cr_2_CT_x_ and Cr_2_NT_x_ at ambient temperature with OH and F groups present [114]. Surprisingly, Mn_2_NT_6_ remains ferromagnetic independently of surface modifications [115]. It should be noted that the stated magnetic moment properties are as yet simply theoretical predictions and have not been observed experimentally. This is because it is difficult to synthesize MXene compounds (particularly pure ones) and there is little control over the surface chemistry [116].

### 3.5. Optical Properties

Devices that utilize photocatalysis, photovoltaics, optoelectronics, or clear conductive electrodes benefit greatly from an electrode material’s ability to absorb both visible and ultraviolet light. Transmittance of up to 86% was observed for films with a thickness in the range of 5–70 nm of Ti_3_C_2_T_x_ [117], and this film absorbed light with a wavelength between 300 and 500 nm. Furthermore, depending on the film thickness, it may exhibit a prominent and wide absorbance peak at about 700–800 nm, resulting in a light-greenish film hue [118] and is significant for photothermal treatment (PTT) applications. Moreover, the transmittance values may be adjusted by altering the diameter and embedded ions of the material [119]. Hydrazine, urea, and DMSO decrease the transmittance of Ti_3_C_2_T_x_ films, and tetramethylammonium hydroxide (NMe_4_OH) enhances it from 74.9% to 92%. First conceptual simulations depicted that functional groups affect the optical characteristics of these 2D materials. Oxygen terminations differ from fluorinated and hydroxyl ones. In the visible range, fluoride and hydroxide terminations lower absorption and reflectivity, but in the UV area, all terminations increase reflectivity relative to pure MXene [120]. Recently, lateral size reduction of MXene flakes reduced absorbance [121]. MXenes are prospects for flexible transparent electrodes owing to their optical and electrical properties in the visible range and metallic conductivity, and their strong ultraviolet reflectance suggests anti-ultraviolet ray coating materials. Finally, biological and water evaporation applications benefited from 100% light-to-heat conversion efficiency [95]. To develop MXene applications, luminescence efficiency, emission colors, plasmonic, and nonlinear optical characteristics must be understood [122].

## 4. Application of MXenes in Point-of-Care Testing

MXenes are 2D nanomaterials with distinct composition of elements and substantial electrical, optical and mechanical characteristics [123]. Enhanced electrical conductance, great wettability by water, strong stability, effective surface, easy to produce huge amounts in water, and environment-friendly are the distinctive features of MXenes that give them remarkable application prospects in diagnostic and therapeutic applications. Various approaches utilizing MXenes in biosensing and other fields have been stated. MXenes have surface groups such as hydroxyl or oxygen that make them hydrophilic. It is ideal for biosensor applications because its surface can interact with most biomolecules through noncovalent interactions. It has been found that a variety of MXene compositions are biocompatible and noncytotoxic [124]. According to recent research, with remarkable sensitivity, endurance, sensing range, and detection limits, MXenes and derivatives are currently prominent in the field of biosensing [125]. Biosensing devices are of different types either based on the mechanism of transduction or on the biological signaling mechanism, such as electrochemical, optical, immunosensor, enzyme-based sensors, and nucleic acid-based sensors [126]. Numerous MXene-based sensors have been established for examining biomolecules, and some of the examples are summarized in Table 3. Recent applications of MXene-based sensors for point of care (POC) diagnostics are briefly discussed in this section.

### 4.1. Electrochemical Biosensors

**Figure 6:**
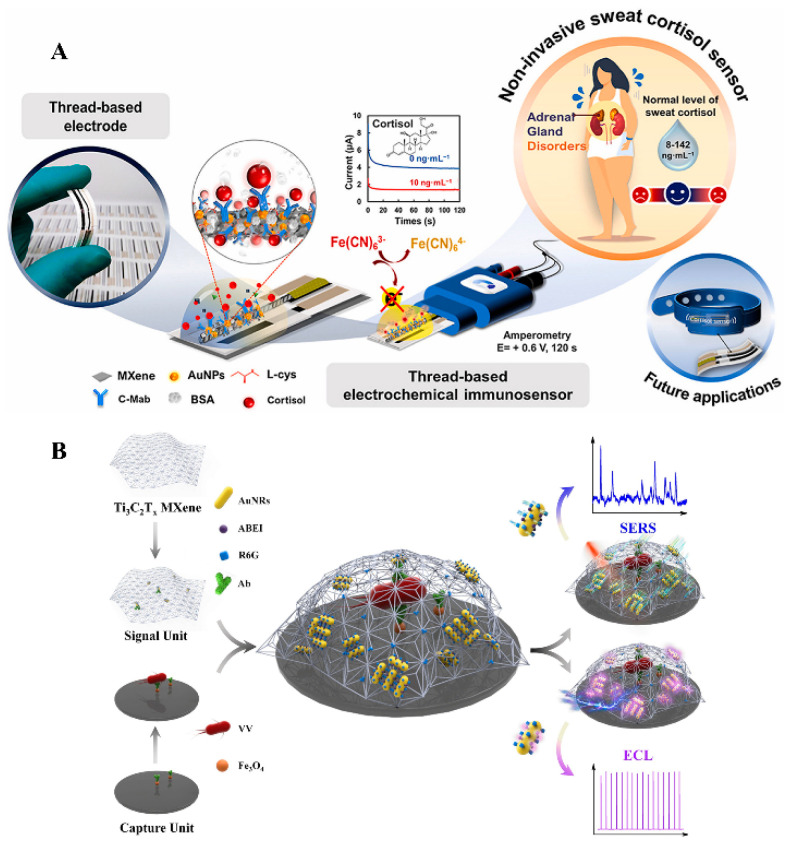
(**A**) Noninvasive electrochemical immunosensor for the detection of cortisol. The thread electrode was modified by immobilization of anti-cortisol on L-cys/AuNPs/MXene; (**B**) Optical sensor based on ECL/SERS fabricated for ultrasensitive detection of *Vibrio vulnificus* (VV), based on a multifunctional MXene material R6G-Ti_3_C_2_T_x_@AuNRs-Ab_2_/ABEI acting as the signal unit. (Reproduced from [163,164] with copyright permission of Elsevier).

Due to their excellent sensitivity, long-term dependability, good precision, speed, low cost, and simplicity of downsizing, electrochemical biosensors have proven valuable for the identification of substances of biological, environmental, agricultural, and therapeutic significance, despite such drawbacks as extensive setup. Electrochemical sensors have advanced significantly with a range of new applications, including single-molecule sensing, in vivo testing, wearable technology, and point-of-care diagnostics [139]. The most often utilized material for making electrochemical sensors among the MXenes is Ti_3_C_2_T_x_. This material’s high conductivity and simple synthesis make it suitable for use as an active component in electrochemical applications [140]. Different types of electrochemical biosensors are available depending on the biomolecules employed to detecting analytes. Figure 6A represents the graphical abstract for the detection of cortisol in sweat.

#### 4.1.1. Enzyme-Based Electrochemical Biosensors

Entrapping enzymes in two-dimensional, multilayered MXene nanolayers with a large surface area can give them a safe microenvironment where they can keep up their activity and stability. MXenes are suitable for entrapping enzymes because of their biocompatibility, metallic conductivity, and hydrophilic surface. Several enzymatic sensors are made by entrapping enzymes such as acetylcholinesterase, glucose oxidase, lactate oxidase, cholesterol oxidase, horseradish peroxidase, tyrosinase, and xanthine oxidase. The manufacturing of Chit/ChOx/Ti_3_C_2_T_x_ using a continuous self-assembled technique resulted in the realization of a voltametric cholesterol sensor. The lipase enzyme was immobilized on chitosan and MXene in this, increasing the electrical conductivity and, consequently, the rate at which electrons transferred. In a different investigation, lipase was immobilized covalently onto Ti_3_C_2_T_x_ MXene. The obtained immobilized lipase was highly reusable and displayed excellent thermal and pH stability [141]. The need for ultrasensitive cholesterol detection at high temperatures was met by immobilizing cholesterol oxidase on MXene/sodium alginate/silica@n-docosane hierarchical microcapsules as a thermoregulatory electrode material for electrochemical biosensors. When compared to existing cholesterol biosensors without a PCM, the newly developed biosensor had a higher sensitivity of 4.63 µAµM^−1^cm^−2^ and a lower limit of detection of 0.081 µM at elevated temperatures, providing exceptional and reliable cholesterol detection for real biological fluids over a broad temperature range [142]. Regarding lactate oxidase (Lox) mounted on Ti_3_C_2_@Eu-SnO_2_, the spectroscopy results show that Eu-SnO_2_, Ti_3_C_2_, and Lox exhibit high hybrid coupling and compatible with biological molecules. A strong linearity in the lactate concentration is revealed by the enzymatic electrochemical biosensor built using Ti_3_C_2_@Eu-SnO_2_/Lox on glassy carbon electrode (GCE). Its low detection limit is 3.38 × 10^−10^ mol L^−1^, with a high sensitivity of 4.815 mA nmol^−1^ L cm^−2^. Furthermore, the created biosensor can accurately monitor lactate in serum samples with significant efficiency [143]. A sensitive enzymatic glucose detection biosensor based on surface-functionalized MXene (Ti_3_C_2_T_x_) has been reported. The biosensor makes use of the functionalized MXene’s high electrical conductivity and many active sites to provide a transfer channel for the electrons produced by the redox interaction between glucose and the enzyme glucose oxidase (GOx). High sensitivity is displayed by the sensor, reaching 5.1 A/A for 10 mM glucose [144]. PB/Ti_3_C_2_T_x_/GOx and Nafion were successfully used to create a unique paper-based screen-printed ionic liquid–graphene device to detect glucose in actual plasma samples. This platform could be utilized for direct measurement and used to track other processes that result in the production of H_2_O_2_ as a by-product [145].

#### 4.1.2. Electrochemical Immunobiosensors

Many biological recognition devices for protein detection fall within the category of electrochemical immunosensors. Electrochemical immunosensors made using MXene and its compounds have been used to detect a number of cancer biomarkers as well as cardiovascular disease biomarkers, sweat biomarkers, inflammatory biomarkers, and others [146]. In a recent study, an immunoassay that uses platelet membrane–Au nanoparticles–delaminated V_2_C nanosheets as the substrate of the sensing interface and methylene blue–aminated metal organic framework (MB@NH_2_-Fe-MOF-Zn) as an electrochemical signal probe was created. To improve the electrochemical sensing performance, the biosensor effectively integrates the exceptional loading property of NH_2_-Fe-MOF-Zn with the high ionic conductivity of AuNPs-loaded V_2_C MXene. The acquired antifouling biosensor has great efficacy for CD44 analysis with a linear range of 0.5 ng mL^−1^ to 500 ng mL^−1^ and is capable of highly sensitive and selective screening of CD44 and CD44-positive tumor cells in heterogeneous liquids [147].

#### 4.1.3. Nucleic Acid-Based Biosensors

Nucleic acid offers good detection capabilities because it is a biomolecule that is stable and simple to handle. Combining the benefits of nucleic acid probes and electrochemical detection, nucleic acid-based electrochemical biosensors enable the sensitive detection of analytes such as genetic material, peptides, cellular structures, inorganic compounds, and cells. Aptamer-based electrochemical biosensors are simple, trustworthy, quick to react, inexpensive, and have tolerable reproducibility. A lead-specific binding DNA molecule was used as the molecular identification molecule on an electrode modified with Au nanoparticles and Nb_4_C_3_Tx to create an electrochemical aptasensor for the superior selectivity and sensitive detection of lead. Through the AuS bond, the Au@Nb_4_C_3_Tx is coupled with lead-binding DNA that has undergone thiol modification. With a limit of detection and linear range of 4 nM and 10 nM to 5μM respectively, the DNA-Au@Nb_4_C_3_Tx-modified glassy carbon electrode demonstrated superior selectivity and improved specificity towards the detection of lead. This research has demonstrated the viability of employing Nb_4_C_3_T_x_ as a reliable immobilization platform for DNA nucleotides in a variety of biological and environmental sensing applications [148]. The biosensor for gliotoxin detection outperformed previously established sensors in terms of great selectivity, good repeatability, and acceptable stability with an LOD of 5 pM in real samples. With regard to clinical applications, this discovery presents a novel route for mycotoxin detection employing MXenes and DNA nanostructure [149]. An aptasensor based on Ti_3_C_2_T_x_@FePcQDs that exhibits good sensing properties in human serum has been created. It has a detection limit of 4.3 aM and a wide linear range of miRNA-155 concentrations from 0.01 fM to 10 pM. The generated Ti_3_C_2_T_x_@FePcQDs-based aptasensor had shown a number of benefits, including successful cDNA immobilization and cross-linking of cDNA/miRNA-155, as well as the ability to prepare a sensing system without the need for labeled probes or electrochemical indicators [150]. The N gene of SARS-CoV-2 was effectively detected with a highly sensitive, quick, and selective Ti_3_C_2_T_x_ biosensor that was functionalized with DNA primers. With ssDNA/Ti_3_C_2_T_x_ sensors, a distinct differentiable response to the N gene of SARS-CoV-2 may be shown at a concentration as low as 105 copies/mL in synthetic saliva, which is within the current detection limit of traditional qPCR testing. This study demonstrates the viability of creating real-time, extremely reliable diagnostic tools for clinical tests based on DNA-functionalized Ti_3_C_2_T_x_ MXenes under the present COVID-19 outbreak [151].

### 4.2. Optical Biosensors

MXenes possess special qualities that make it easier to create optical biosensors with the highest performance. MXenes have favorable energy levels and a broader absorption band, which make them excellent candidates for optical, photothermal, and photoelectrochemical biosensing despite light interference. Figure 6B presents an ECL/SERS-based optical biosensor for the detection of *Vibrio vulnificus*. The applications of MXenes in optical biosensing will be outlined in the following section, with a focus on some notable recent examples in photoluminescence, electrochemiluminescence, and photoelectrochemistry [152].

#### 4.2.1. Photoluminescent Biosensors

Fluorescence analysis has a high sensitivity for the identification of biomolecules. Two methods are generally recommended for creating MXene-based fluorometric sensors. For “on/off” effects, the first type employs MXene nanostructures as efficient quenchers (donors) for fluorophores or other fluorescent nanomaterials (donors), such as dyes, quantum dots, and metal nanoparticles. The second type employs luminescent magnetic quantum dots as the signal output modules, which are effectively and promptly quenched by the presence of samples. Various design methodologies can be used to create fluorescent biosensors based on MXenes with a variety of characteristics [153]. Nitrogen-doped Ti_3_C_2_ QDs with a high photoluminescence quantum yield (PLQY) have been used to create an attractive mediator-free biosensor with high sensitivity and a detection limit of up to 100 µM for the detection of H_2_O_2_ [154]. Ti_3_C_2_ nanosheets and red-emitting carbon dots (RCDs) were coupled to create a potent and focused fluorescent turn-on nanosensor for glucose sensing. Ti_3_C_2_ nanosheets were able to effectively quench (>96%) the fluorescence intensity of RCDs (IFE). The nanosensor can be used to monitor glucose based on hydrogen peroxide generated by the oxidation of glucose catalyzed by glucose oxidase. The detection limit under ideal circumstances was 50 μM (S/N = 3) [155]. An Ag@Ti_3_C_2_–MXene nanohybrid was used to create a fluorescent turn-on detection system, which demonstrated biosensing qualities for the recognition of neuron-specific enolase (NSE), with great sensitivity (~771 ng mL^−1^), a wider linear sensing range (0.0001–1500 ng mL^−1^), finer detection limit (0.05 pg mL^−1^), and a quicker reaction within 12 min [156].

#### 4.2.2. Electrochemiluminescence Biosensors

One of the most popular methods for making electrochemically stimulated ECL emitters emit light is electrochemiluminescence (ECL), sometimes known as electrogenerated chemiluminescence [157]. A potent instrument in the realm of biosensing, ECL has drawn a lot of interest. To detect HIV from serum samples with a LOD of 0.3 fM, a Ti_3_C_2_T_x_ MXenes altered ZIF-8 aptamer-based sensor was created [158]. Based on PEIRu@Ti_3_C_2_@AuNPs ECL material, a biosensor for the RdRp gene has been created. The ssDNA on the surface of the biosensor is cut by activated CRISPR-Cas12a, which also makes the ferrocene reformed at one end of the DNA to shift away from the top of the electrode, boosting the ECL signal. The magnitude of the electrochemiluminescence change reflects the amount of the target gene present. This method encourages the medical use of ECL biosensors based on CRISPR-Cas12a and innovative complex materials and adds to the quick and suitable sensing of SARS-CoV-2-associated nucleic acids with a limit of detection of 12.8 aM [159].

#### 4.2.3. Photoelectrochemical Biosensors

Photoelectrochemical (PEC) biosensing has garnered a lot of interest due to its capacity to identify biomolecules using the photocurrent produced by biomolecule oxidation. PEC is a promising cost-effective technique to alter chemical energy to electricity when applied voltage and light illumination are present [160]. Using Ti_3_C_2_T_x_ and paper-thin covalent organic framework nanosheets (referred to as TTPA-CONs), new composites were created that had PEC-sensing capabilities. With great sensitivity, a detection limit of 0.0003 ng/mL, and exceptional stability, the produced TTPA-CONs/Ti_3_C_2_T_x_ complex can be utilized as photocathodes for PEC sensing of prostate-specific antigen (PSA), [161] built a PEC/EC sensing platform using a MIP functionalized Bi_2_S_3_/Ti_3_C_2_T_x_ MXene nanocomposite and achieved the dual-signal detection of chlorogenic acid (CGA). Bi_2_S_3_/Ti_3_C_2_T_x_ MXene’s superior photoelectric conversion efficiency not only produced a PEC signal with a low background but also had electrocatalytic properties. By merging a molecular imprinting technique with Bi_2_S_3_/Ti_3_C_2_T_x_ MXene as the photoactive material and CGA as the idea target, a quick and extremely sensitive PEC sensor was created [162].

### 4.3. Wearable Biosensors

**Figure 7:**
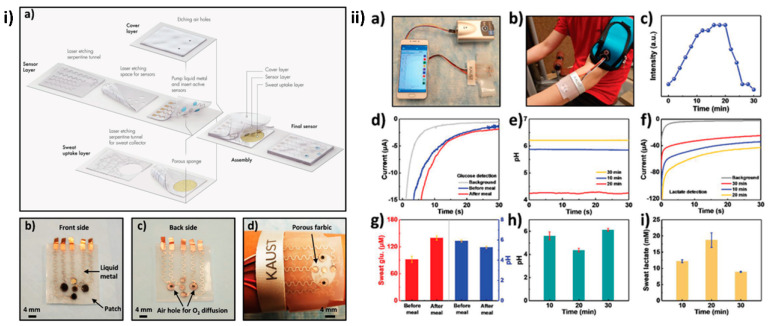
(**i**) (**a**) Representation of the patch system of a sensor that consists of a cover layer, a sweat-uptake layer, and a sensor layer. (**b**) Front-side optical picture of the pH sensor (bottom), reference electrode (top), counter electrode (middle), and sensor array (left and right). (**c**) An optical picture of the sensor array’s back side. (**d**) Photographs of the biosensor band printed optically on a person’s skin. (**ii**) On-body sensor patch pH, lactate, and glucose monitoring. (**a**) A portable electrochemical analyzer powers and operates the sweat-monitoring patch and communicates with commercial mobile phones through Bluetooth. (**b**) A skin-mounted electrochemical analyzer connects to the sweat-monitoring patch. (**c**) On-body cycling resistance profile. (**d**) Measured chronoamperometric responses of three glucose sensors and pH changes before and after meals. (**e**) Measured pH sensor levels throughout workout. (**f**) Lactate sensor chronoamperometry during exercise. (**g**) Three glucose and pH sensors compared before and after meals. (**h**) Three pH sensor comparisons during the workout. (**i**) Lactate sensor comparisons during exercise. (Reprinted from [167] with copyright permission from John Wiley and Sons).

Electrically conductive and highly flexible nanosheets make up typical MXene films. Even when the structure is mechanically deformed, the horizontal stacking allows for the creation of a variety of electrical pathways, and the interaction between the negatively charged MXene surface and the positively charged elements present in between or dipolar water molecules can help preserve the structure. Due to these benefits, MXenes are appropriate for applications involving wearable sensors [165]. An electrochemical portable patch system was fabricated for the detection of glucose and lactate in sweat samples (Figure 7). Ti_3_C_2_T_x_-based MXenes of great purity were prepared by in situ hydrofluoric acid wet etching. PVDF membrane was coated with Ti_3_C_2_T_x_ via vacuum-assisted filtering. The Ti_3_C_2_T_x_-coated PVDF membrane was utilized to construct a wearable pressure sensor that monitored finger bending/stretching resistance. Health monitoring with Ti_3_C_2_T_x_-coated PVDF membrane strain sensors appear promising [166].

A stretchable piezoresistive pressure detector based on a Ti_3_C_2_T_x_ nanosheet-dipped polyurethane sponge has been made from a molybdenum microstructured electrode produced by helium plasma irradiation. This electrode-modifying method allows the easy conversion between sponge deformation and MXene interlamellar displacement, resulting in elevated sensitivity (1.52 kPa^−1^) and strong linearity (r^2^ = 0.9985) in a broad sensing range (0–100 kPa) with a pressure detection response time of 226 ms. The flexible pressure sensor can also sense human radial pulse, monitor finger tapping, foot movement, and identify objects, making wearable biomonitoring and health evaluation feasible. Epidermal sensors made of hydrogel can be employed in electronic skins, soft robotics, and personal health-care monitoring. A flexible and wearable epidermal sensor made of MXene/polyampholytes hydrogel has been created to monitor the daily activity of ADHD patients [168].

## 5. Conclusions and Future Prospects

MXenes have shown tremendous potential in the domain of sensing due to their superior electrical and optical properties compared to traditional 2D materials. The number of investigations on MXene is increasing drastically, and we are hopeful that the majority of concerns may be resolved by MXene research following the appropriate path. In this review, the various design and synthesis method for fabricating MXenes, such as hydrothermal, acid etching, etc., have been discussed and their applications are listed with examples in order to serve as a roadmap for future efforts in the creation of efficient sensing platforms. Furthermore, MXene possess exceptional electronic, magnetic, optical and thermal properties that are superior to other 2D materials such as graphene and TMD, and hence MXenes are subsequently used for applications in developing sensing devices. Additionally, MXene shows significant potential in the fabrication of conductive substrates for different electrode systems, particularly patterned electrodes. This opens the door to the creation of a wide range of improved electrochemical sensing technologies, including portable and wearable sensing devices for noninvasive bodily fluid tracking and tiny monitoring equipment for practical interpretation. In a nutshell, MXene’s usefulness as a dependable electrochemical sensing device has been demonstrated by overcoming several challenges, and this trend is predicted to continue in the future. As a result, we are optimistic that MXene will fulfill its full potential simply by bringing 2D materials to commercial applications.

## Figures and Tables

**Table 1:** Methods used for the synthesis and functionalization of MXene.

Material	Synthesis Protocol	Reference
MXene/NiFe_2_O_4_ nanocomposites	One step hydrothermal	[49]
NiCo-LDH/MXene hybrids	Heterojunction surface	[50]
2D Ti_2_CT_x_ MXene	HF etching	[51]
Ti_3_C_2_T*_z_* MXene	Intensive layer delamination and acid	[52]
Cr_2_CT_x_ MXene	Etching	[53]
MXene-derived nanoflower-shaped TiO_2_@Ti_3_C_2_	Heterojunction (In situ Transformation)	[54]
Mo_2_CT_x_ MXene	Mo_2_Ga_2_C by etching	[55]
Ti_3_C_2_T_x_ MXene/graphene nanocomposites	Hydrothermal method	[56]
Ti_3_C_2_-MXene/ZIF-67/CNTs heterostructure	heterojunction	[57]
MXene hybrids	Heterojunction	[58]

Reproduced from [59] with copyright permission of Elsevier.

**Table 2:** Comparison of properties of MXenes with other 2D nanomaterials.

Properties	MXenes		TMDs (Transition Metal Dichalcogenides)		Graphene	
	Comments	References	Comments	References	Comments	References
Conductivity	9880 S/cm of Ti_3_C_2_T_x_	[65]	5.0 S/cm of MoS_2_	[66]	106 S/cm of pristine graphene	[67]
Functionalization	Abundant hydrophilic terminations for easy functionalization	[68,69]	Lacking dangling bonds or π electrons for covalent linking	[70,71]	Lacking surface terminations for biofunctionalization	[72,73]
Dispersity	Stable water dispersity	[74]	Easy to form agglomerates	[70]	Intense aggregation of pristine graphene in water	[75]
Bandgap	Metallic bandgap of Ti_3_C_2_, could be tunable by terminations and intercalations	[76,77,78]	1.8 eV of monolayer MoS_2_, 1.45 eV of monolayer WS_2_	[79]	0 of bilayer graphene	[79]
Biosafety	Good biocompatibility, negligible cytotoxicity	[80,81]	Few cytotoxic responses of TMDs in cells	[82,83]	Low cytotoxicity and good biocompatibility	[77,84]
Stability	Vulnerable in humid, oxygen-enriched environment	[85]	Grave degradation in ambient oxygen and moisture	[86]	Rather stable in ambient conditions	[87,88]
Distinctive merits in biosensing	Wide adsorption spectrum for optical sensing; strong chelation interaction with DNA	[89,90]	Formation of Au–S bonds with gold-based nanomaterials	[91,92]	Superior catalysis and fast charge transfer	[93]

Reproduced from [94] with copyright permission of Elsevier.

**Table 3:** Some of the MXene-based sensors.

Type of Biosensor	Formulation	Analyte	Sensing Range	Limit of Detection (LOD)	Reference
Electrochemical biosensor	Prussian blue/Ti_3_C_2_ MXene	Exosomes secreted by various cancer cells	5 × 10^2^–5 × 10^5^ particles µL^−1^	229 particles µL^−1^	[127]
	MXene–MoS_2_	MicroRNA-21 biomarker for cancer diagnosis and prognosis	100 fM to 100 nM	26 fM	[128]
	MXene @Au NPs@ methylene blue	Prostate-specific antigen	5 pg mL^−1^ to 10 ng mL^−1^	0.83 pg mL^−1^	[129]
	MXene-based cytosensor	HER2-positive cancer cells	10^2^–10^6^ cells mL^−1^	47 cells mL^−1^ (Total detection time of ~75 min)	[130]
	MXene–graphene	Influenza A (H1N1) virus	125–250,000 copies mL^−1^	125 copies mL^−1^	[131]
		SARS-CoV-2	1 fg mL^−1^–10 pg mL^−1^	1 fg mL^−1^ (Average response time for both virus ~50 ms)	
Optical biosensor	MXene–Au	Gram-negative and Gram-positive bacteria	3 × 10^5^–3 × 10^8^ CFU mL^−1^	3 × 10^5^ CFU mL^−1^	[132]
	Ti_3_C_2_T_x_ MXene–Au NPs@polyimide thin film	Carcinoembryonic antigen	0.1–100 ng mL^−1^	0.001 ng mL^−1^	[133]
	MXene N-Ti_3_C_2_ quantum dot/Fe^3+^	Glutathione	0.5–100 × 10^−9^ fM	0.17 × 10^−9^fM	[134]
	MXene-derived quantum dot@Au	Triple-negative breast cancer	5 fM to 10 nM,	1.7 fM	[135]
	MXene-CRISPR-Cas 12a	Siglec-5	20 fM–100 pM	20.22 fM	[136]

Reproduced from [137,138] with copyright permission from Elsevier.

## Data Availability

Not applicable.
